# A Review of Guidelines for the Use of Tranexamic Acid in the Management of Postpartum Hemorrhage and Assessment of Associated Maternal Outcomes

**DOI:** 10.7759/cureus.116511

**Published:** 2026-09-18

**Authors:** Khushi Shekhawat, Akanksha Ahuja, Ranya Parekh, Arun Nair

**Affiliations:** 1 School of Medicine, St. George's University, St. George’s, GRD; 2 School of Medicine, Hofstra University/Donald and Barbara Zucker, Hempstead, USA; 3 Department of Pediatric Hematology Oncology, Cohen Children's Medical Center, New Hyde Park, USA

**Keywords:** bleeding risk, efficacy of tranexamic acid, guideline, obstetrics and gynecology, postpartum hemorrhage

## Abstract

Postpartum hemorrhage (PPH) remains a major cause of maternal morbidity and mortality worldwide, requiring rapid recognition and coordinated hemostatic intervention. Tranexamic acid (trans-4-aminomethylcyclohexane-1-carboxylic acid; TXA), an antifibrinolytic agent that inhibits plasminogen-mediated fibrin degradation, has emerged as an important component of PPH management. Despite growing evidence supporting TXA, recommendations on its timing and placement within PPH treatment algorithms vary across major international guidelines. This narrative review examines the pharmacology, clinical indications, safety profile, and therapeutic role of TXA in PPH. It evaluates evidence supporting TXA in obstetric and non-obstetric hemorrhage, compares its role with other hemostatic therapies, and critically examines recommendations from major international and national organizations. It pays particular attention to differences in how these guidelines position TXA within PPH treatment pathways and integrate it with other first-response interventions. This review also considers uncertainties around prophylactic use, alternative hemostatic therapies, and variation in guideline implementation. Greater consistency in evidence-based recommendations may clarify TXA's role in contemporary PPH management while allowing flexibility for differences in healthcare settings and resources.

## Introduction and background

Postpartum hemorrhage (PPH) remains a leading cause of maternal mortality worldwide and is associated with substantial maternal morbidity [1]. Traditional definitions of PPH commonly use blood-loss thresholds, including 500 mL or more after vaginal delivery and 1000 mL or more after cesarean delivery [2]. The American College of Obstetricians and Gynecologists (ACOG) subsequently adopted a unified definition of cumulative blood loss of 1000 mL or more, or blood loss accompanied by signs or symptoms of hypovolemia within 24 hours following the birth process, including intrapartum blood loss, regardless of route of delivery [1]. More recently, the 2025 World Health Organization (WHO) consolidated guidelines recommend initiating first-response treatment at objectively measured blood loss of 300 mL or more accompanied by any abnormal hemodynamic sign or at blood loss of 500 mL or more, whichever occurs first within 24 hours after birth [3].

PPH affects millions of women annually and causes nearly 45,000 maternal deaths worldwide [3]. PPH remains an important cause of severe maternal morbidity in the United States, where its incidence has increased over time [1,4]. PPH is commonly categorized using the Four Ts: tone (uterine atony), tissue (retained placental tissue), trauma (genital tract injury, uterine injury, or inversion), and thrombin (coagulopathy), with uterine atony accounting for most cases [1,2]. Risk factors for uterine atony include uterine overdistension from multiple gestation, polyhydramnios, or fetal macrosomia, prolonged or augmented labor, chorioamnionitis, and high parity [1].

Managing PPH requires rapid identification of the bleeding source and coordinated medical, resuscitative, mechanical, and surgical interventions [1,3]. Initial evaluation focuses on identifying the underlying etiology using the Four Ts framework [1,2]. Uterotonics, particularly oxytocin, remain central to treating atony-associated PPH, with additional agents such as carboprost, ergometrine, and misoprostol selected based on clinical circumstances and contraindications [1,3]. Tranexamic acid (trans-4-aminomethylcyclohexane-1-carboxylic acid; TXA) provides an antifibrinolytic approach to hemorrhage control, and current WHO guidance includes TXA in an immediate first-response treatment bundle alongside uterine massage, oxytocic therapy, intravenous (IV) fluids, genital tract examination, and escalation of care when required [3]. Persistent hemorrhage may require intrauterine tamponade, uterine compression sutures, arterial embolization, or hysterectomy depending on the etiology, severity of bleeding, response to initial therapy, and available resources [1].

Severe or uncontrolled PPH can result in hemorrhagic shock, disseminated intravascular coagulation, acute kidney injury, loss of fertility following peripartum hysterectomy, and hypopituitarism secondary to pituitary ischemic injury (Sheehan syndrome) [1]. The World Maternal Antifibrinolytic (WOMAN) Trial demonstrated a reduction in death due to bleeding among women with PPH treated with TXA, with the greatest benefit observed when treatment was initiated within three hours of delivery [5]. Despite an expanding evidence base, international guidance on diagnosing and managing PPH remains heterogeneous [3]. Differences in recommendations regarding the timing and positioning of TXA within treatment algorithms therefore warrant focused comparison. This narrative review describes the pharmacology and clinical indications of TXA, critically compares international guidelines governing its use in PPH, evaluates the clinical evidence supporting TXA relative to other hemostatic therapies, and assesses associated maternal outcomes to identify opportunities to standardize evidence-based TXA use in obstetric hemorrhage.

## Review

Methods

This narrative literature review examined TXA use in PPH, with particular emphasis on current clinical guidelines, maternal outcomes, and TXA's role relative to other hemostatic therapies. The study team defined the review's scope and objectives before evaluating the literature and developing the manuscript. The team identified relevant literature by searching PubMed and Google Scholar. Search terms included "postpartum hemorrhage", "tranexamic acid", "antifibrinolytic therapy", "maternal outcomes", "WOMAN Trial", "postpartum hemorrhage guidelines", and terms related to alternative hemostatic therapies. Search terms were combined using Boolean operators (AND/OR) as appropriate. We prioritized English-language human studies relevant to PPH management, the efficacy or safety of TXA, maternal outcomes, or international recommendations for TXA use. We considered major clinical trials, systematic reviews, and guidance from relevant professional and international organizations, including the WHO, ACOG, the Society of Obstetricians and Gynaecologists of Canada (SOGC), and the Royal College of Obstetricians and Gynaecologists (RCOG), where applicable, as they represent major international or national professional organizations whose recommendations allowed comparison of TXA use across distinct obstetric practice frameworks. Because this study was a narrative review rather than a systematic review, we did not apply a predefined search period, a formal record-level screening protocol, or a standardized risk-of-bias assessment. We evaluated selected literature for relevance to the predefined objectives and distributed sections among team members for analysis and drafting. Drafted sections subsequently underwent cross-review to assess scientific accuracy, consistency of interpretation, citation support, and coherence across the manuscript. The study team then reviewed and approved the completed manuscript before submission.

TXA: pharmacology and clinical profile

TXA is a synthetic lysine analog with antifibrinolytic properties, developed in the early 1960s by Japanese researchers Shosuke and Utako Okamoto [6]. Its clinical applications subsequently expanded across multiple hemorrhagic conditions, including heavy menstrual bleeding, inherited bleeding disorders, surgical bleeding, trauma, and obstetric hemorrhage [6]. TXA exerts its antifibrinolytic effect primarily by reversibly blocking lysine-binding sites on plasminogen, reducing its interaction with fibrin and subsequent fibrinolysis [6,7]. Under physiological conditions, plasminogen activators convert plasminogen to plasmin, which promotes fibrin degradation [7]. By interfering with plasminogen binding to fibrin, TXA limits fibrin degradation and stabilizes existing hemostatic clots rather than directly initiating coagulation [6,7]. This mechanism provides the pharmacologic basis for its use during acute hemorrhage, when excessive fibrinolytic activity may contribute to continued clot breakdown.

At recommended doses, TXA is generally well tolerated [6,7]. Common adverse effects include gastrointestinal (GI) symptoms, particularly nausea and diarrhea [7]. Because TXA is predominantly eliminated renally, dose reduction may be necessary in patients with impaired renal function [6]. Important contraindications include active venous or arterial thrombosis and hypersensitivity to TXA, and clinicians should also consider patient-specific thrombotic and neurologic risk factors when determining treatment [6]. Seizures represent an important dose-related safety consideration with TXA [8]. Most reported TXA-associated seizures have occurred following cardiac procedures, where relatively high-dose regimens have historically been used, although seizures have also been reported outside the cardiac surgical setting [8]. Experimental and clinical evidence suggests that TXA can reduce inhibitory neurotransmission by antagonizing glycine and gamma-aminobutyric acid type A receptors, providing a plausible mechanism for its proconvulsant effects at sufficiently high concentrations [8]. A meta-analysis of 16 studies involving 45,235 adults undergoing cardiac surgery found that TXA exposure was associated with an increased risk of postoperative seizures [9]. In contrast, the WOMAN Trial, which evaluated 1 g IV TXA in more than 20,000 women with PPH, did not demonstrate a significant increase in seizures or thromboembolic complications compared with placebo [5]. These findings highlight the importance of distinguishing safety signals associated predominantly with higher-dose perioperative regimens from those observed with recommended obstetric dosing.

Clinical evidence for TXA in hemorrhage

Large-scale randomized evidence supporting TXA in acute major hemorrhage was established in the non-obstetric setting by the CRASH-2 Trial, which randomized 20,211 adult trauma patients with significant bleeding or risk of significant bleeding and demonstrated reductions in both all-cause mortality and death due to bleeding with TXA [10]. A subsequent exploratory analysis demonstrated a time-dependent treatment effect: TXA reduced death due to bleeding when administered within one hour of injury and between one and three hours, whereas administration beyond three hours was associated with increased bleeding mortality [11]. Evidence from surgical populations further demonstrated that TXA reduces transfusion requirements. A systematic review and meta-analysis of 129 randomized trials involving 10,488 surgical patients found that TXA reduced the probability of receiving an allogeneic blood transfusion. However, its effects on thromboembolic events remained uncertain [12].

The pivotal evidence supporting TXA for treating PPH came from the WOMAN Trial. This international, randomized, double-blind, placebo-controlled trial enrolled 20,060 women with clinically diagnosed PPH across 193 hospitals in 21 countries and compared 1 g IV TXA with placebo in addition to usual care [5]. The primary composite outcome of death from all causes or hysterectomy was not significantly reduced. However, death due to bleeding occurred in 1.5% of women receiving TXA compared with 1.9% receiving placebo (RR 0.81; 95% CI 0.65-1.00), with a greater reduction among women treated within three hours of delivery (1.2% vs. 1.7%; RR 0.69; 95% CI 0.52-0.91) [5]. The trial did not demonstrate a significant increase in thromboembolic events with TXA [5].

Treatment timing is particularly important when translating these findings into clinical practice. In the WOMAN Trial, the three-hour interval was measured from delivery rather than from the recognition or diagnosis of PPH [5]. Consequently, delays in recognizing hemorrhage reduce the remaining period during which TXA has demonstrated clinical benefit. TXA was administered therapeutically after PPH had been diagnosed rather than routinely before hemorrhage occurred [5]. These findings support administering TXA as soon as possible after recognizing PPH, rather than delaying it until prior interventions have failed [3,5]. Consistent with this evidence, the WHO recommends IV TXA as soon as possible and within three hours of birth for women with PPH following either vaginal or cesarean birth and does not support administration beyond three hours after birth [3].

The E-MOTIVE Trial further evaluated the importance of early recognition and coordinated treatment in secondary-level hospitals in Kenya, Nigeria, South Africa, and Tanzania [13]. The intervention combined objective blood-loss assessment using a calibrated collection drape with a first-response treatment bundle consisting of uterine massage, oxytocic therapy, TXA, IV fluids, genital tract examination, and escalation of care, supported by an implementation strategy [13]. The primary composite outcome of severe PPH, laparotomy for bleeding, or maternal death from bleeding occurred in 1.6% of patients in the intervention group compared with 4.3% receiving usual care (RR 0.40; 95% CI 0.32-0.50) [13]. Because E-MOTIVE evaluated a multicomponent detection-and-treatment strategy rather than TXA in isolation, this effect cannot be attributed specifically to TXA. Instead, the trial supports rapid recognition of PPH and coordinated implementation of evidence-based first-response interventions.

The 2025 WHO consolidated guideline was developed with access to a broader, more recent evidence base on TXA and coordinated first-response treatment [3]. Evidence regarding prophylactic TXA is less definitive. In the TRAAP Trial, 4,079 women with planned vaginal delivery were randomized to receive 1 g IV TXA or placebo in addition to prophylactic oxytocin; among women who underwent vaginal delivery, measured blood loss of at least 500 mL occurred in 8.1% with TXA and 9.8% with placebo, a difference that did not reach statistical significance (RR 0.83; 95% CI 0.68-1.01; p = 0.07) [14]. The WOMAN-2 Trial similarly found no reduction in clinically diagnosed PPH among women with moderate or severe anemia receiving prophylactic TXA before vaginal birth (7.0% vs 6.6%; RR 1.05; 95% CI 0.94-1.19) [15]. Accordingly, WHO does not recommend routine TXA for prevention of PPH at either vaginal or cesarean birth [3]. However, a 2024 individual-patient-data meta-analysis of five high-quality randomized trials involving 54,404 women found that TXA reduced life-threatening postpartum bleeding (0.65% vs 0.85%; OR 0.77; 95% CI 0.63-0.93), without evidence that the effect varied according to baseline bleeding risk or whether TXA was administered before or after PPH diagnosis [16]. The investigators did not support routine administration to all women but suggested consideration before PPH diagnosis in women at high risk of death from bleeding [16]. Thus, current evidence strongly supports early TXA for established PPH, whereas its role before PPH diagnosis remains less certain and may warrant further evaluation in selected high-risk populations.

Comparative roles of hemostatic therapies in PPH

TXA occupies a distinct role within the hemostatic management of PPH because it has a broadly applicable, evidence-supported role in the early treatment of established hemorrhage [3,5]. Other hemostatic therapies should not be regarded as interchangeable comparators because their indications, mechanisms, and clinical applications differ substantially. Table 1 compares different hemostatic therapies in the management of PPH.

**Table 1:** Comparative roles of hemostatic therapies in PPH DDAVP: desmopressin, EACA: ε-aminocaproic acid, PPH: postpartum hemorrhage, rFVIIa: recombinant activated factor VII, TXA: tranexamic acid, WHO: World Health Organization

Therapy	Principal hemostatic action	Potential role in PPH	Principal limitation
TXA	Inhibits fibrinolysis [6,7]	Early adjunctive treatment of established PPH; recommended as soon as possible and within three hours of birth [3,5]	Clinical benefit is time-dependent [3,5]
EACA	Lysine-analog antifibrinolytic	Pharmacologically related to TXA	No randomized evidence identified for treatment of primary PPH [17]
Fibrinogen replacement	Replaces fibrinogen during hemorrhage-associated coagulopathy	Fibrinogen concentrate or cryoprecipitate may be used when fibrinogen deficiency is present or suspected; WHO identifies ≥2 g/L as a therapeutic target [3]	Indication is dependent on the presence or likelihood of fibrinogen deficiency
rFVIIa	Promotes thrombin generation and clot formation	Potential specialized role in severe PPH unresponsive to initial therapy [18]	Limited evidence base; thromboembolic safety concerns; effectiveness remains uncertain in WHO guidance [3,18]
DDAVP	Promotes release of endogenous von Willebrand factor and factor VIII	May be relevant when obstetric bleeding occurs in selected patients with a DDAVP-responsive inherited bleeding disorder [19]	Not routine PPH therapy; response is disorder-dependent, and hyponatremia and subtype-specific limitations restrict use [19]

This distinction is clinically important. Large randomized obstetric-specific evidence supports TXA, and early treatment recommendations for established PPH include it [3,5]. In contrast, fibrinogen replacement addresses hemorrhage-associated fibrinogen depletion, and WHO identifies ≥2 g/L as a therapeutic fibrinogen target during massive PPH management [3]. TXA and fibrinogen replacement therefore address different components of impaired hemostasis and may be used within the same resuscitative strategy rather than being considered competing treatments.

The roles of recombinant activated factor VII (rFVIIa) and desmopressin are more restricted. In an open-label randomized trial of 84 women with severe PPH unresponsive to uterotonics, rFVIIa reduced the need for invasive second-line therapies compared with standard care (52% vs. 93%; RR 0.56; 95% CI 0.42-0.76), although two venous thrombotic events occurred among women receiving rFVIIa [18]. Despite these findings, the evidence base is substantially smaller than that supporting TXA, and WHO currently considers the effectiveness of rFVIIa in PPH uncertain [3]. Desmopressin should not be considered routine therapy for obstetric hemorrhage but may be relevant in selected patients with an underlying responsive bleeding disorder. Contemporary von Willebrand disease (vWD) guidelines recommend individualized use according to vWD subtype and responsiveness, with important limitations including lack of efficacy in type 3 vWD, contraindication in type 2B vWD, and the risk of hyponatremia [19].

ε-Aminocaproic acid (EACA) shares the lysine-analog antifibrinolytic mechanism of TXA but lacks comparable PPH-specific evidence; a Cochrane review evaluating antifibrinolytic treatment for PPH identified randomized studies of TXA but no studies of EACA [17]. The available evidence therefore does not justify treating EACA and TXA as equivalent obstetric interventions. More broadly, the absence of head-to-head trials among these therapies should not be interpreted simply as a need to determine which agent is superior, because they address different hemostatic abnormalities and clinical scenarios. A more clinically relevant evidence gap concerns how to select, time, and combine adjunctive hemostatic therapies based on hemorrhage severity, etiology, coagulation abnormalities, and response to initial treatment. Within the available evidence, TXA has the clearest role as a broadly applicable early adjunctive treatment for established PPH; EACA lacks comparable obstetric evidence, while fibrinogen replacement, rFVIIa, and desmopressin occupy more targeted roles defined by the clinical and hemostatic context.

Comparison of international guidelines on TXA use in PPH

Major international guidelines consistently recognize a role for TXA in managing PPH but differ in how explicitly they define its timing and position within the treatment pathway [1,3,20,21]. Interpreting these differences requires considering guideline publication dates because the evidence supporting TXA has evolved substantially, particularly after publication of the WOMAN Trial in 2017 [5]. The clearest difference concerns TXA's position relative to other medical therapy. The 2025 WHO consolidated guideline recommends IV TXA in addition to standard care for women with PPH following either vaginal or cesarean birth [3]. For PPH following vaginal birth, WHO incorporates TXA into a standardized first-response treatment bundle alongside uterine massage, an oxytocic agent, IV fluids, examination of the genital tract, and escalation of care [3]. In contrast, ACOG Practice Bulletin No. 183 recommends considering TXA when initial medical therapy for obstetric hemorrhage fails [1]. The 2022 SOGC guideline recommends TXA in patients with PPH as an adjunct to uterotonic therapy [20]. The current RCOG Green-top Guideline No. 52 recommends considering TXA in PPH management but does not more specifically define its position relative to preceding uterotonic therapy [21].

Recommendations also differ in how specific they are about treatment timing. WHO provides the most explicit recommendation, stating that TXA should be administered as soon as possible and within three hours of birth [3]. This timing is based on the WOMAN Trial, in which the greatest reduction in death due to bleeding was observed among women treated within three hours of delivery [5]. ACOG discusses the same time-dependent evidence and states that earlier administration is likely more effective than delayed treatment. Still, it does not establish the three-hour interval as a defined treatment recommendation [1]. SOGC Guideline No. 431 recommends TXA during active PPH but does not specify administration within three hours of birth [20]. RCOG likewise does not include a three-hour treatment window; importantly, it completed its evidence review before publication of the WOMAN Trial, and the guideline identifies the WOMAN Trial as ongoing at the time of development [21]. The absence of a three-hour recommendation in RCOG guidance therefore reflects, at least in part, the evidence available when the guideline was produced rather than a conflicting interpretation of the subsequent WOMAN Trial findings.

The guidelines also vary in the specificity of TXA dosing instructions. WHO recommends 1 g intravenously over approximately 10 minutes, with a second 1-g dose if bleeding continues after 30 minutes or restarts within 24 hours [3]. SOGC specifies a 1-g IV dose, with repeat administration after 30 minutes when required [20]. RCOG includes TXA 1 g intravenously within its major PPH treatment algorithm but does not provide the contemporary WOMAN-based treatment regimen as a formal recommendation [21]. ACOG discusses the 1 g regimen evaluated in the WOMAN Trial but does not provide a separate prescriptive TXA dosing regimen within its recommendations [1]. Table 2 summarizes the distinctions seen among the aforementioned guidelines.

**Table 2:** Comparison of international recommendations for TXA in PPH ACOG: American College of Obstetricians and Gynecologists, IV: intravenous, PPH: postpartum hemorrhage, RCOG: Royal College of Obstetricians and Gynaecologists, SOGC: Society of Obstetricians and Gynaecologists of Canada, TXA: tranexamic acid, WHO: World Health Organization

Guideline	Recommendation for TXA	Position in treatment	Explicit timing recommendation	Dosing guidance
WHO (2025) [3]	Recommended in addition to standard care for established PPH	Incorporated into first-response treatment; included in standardized treatment bundle for PPH following vaginal birth	As soon as possible and within three hours of birth	1 g IV over approximately 10 minutes; repeat 1 g if bleeding continues after 30 minutes or restarts within 24 hours
ACOG (2017; reaffirmed 2024) [1]	Consider TXA when initial medical therapy fails	Following failure of initial medical therapy	No defined treatment window; acknowledges that earlier administration is likely more effective	WOMAN regimen discussed, but no separate prescriptive TXA dosing recommendation
SOGC (2022) [20]	TXA can be used in patients with PPH as an adjunct to uterotonics	Adjunct to uterotonic therapy	No explicit three-hour treatment window stated	1 g IV; repeat after 30 minutes if required
RCOG (2016) [21]	Consider TXA in the management of PPH	Position relative to preceding uterotonic therapy not specifically defined	No three-hour window specified; guideline predates publication of WOMAN	1 g IV included in major PPH management algorithm

Taken together, the guidelines show greater consistency on the role of TXA than on its timing and placement within PPH treatment algorithms. WHO provides the most explicit current recommendation for early administration and integrates TXA into coordinated first-response treatment, whereas ACOG and SOGC support TXA but differ in how they position it relative to uterotonic therapy [1,3,20]. The current RCOG guideline reflects an earlier evidence era and should therefore be interpreted separately from post-WOMAN guidance [21]. Because the clinical benefit of TXA is time-dependent [5], greater consistency in future guideline recommendations on when to administer TXA may reduce implementation ambiguity. However, direct comparative evidence demonstrating that differences among these guideline frameworks produce differences in maternal outcomes is currently lacking.

Discussion

The principal finding of this review is that major international guidelines consistently recognize a role for TXA in managing PPH but differ in how explicitly they define its timing and position within treatment pathways [1,3,20,21]. This distinction matters given TXA's time-dependent benefit, with the strongest evidence supporting administration as early as possible and within three hours of birth [3,5]. Guideline heterogeneity therefore reflects less on whether TXA has a role in PPH than on when and how to incorporate it into treatment.

Several factors may contribute to these differences. Most importantly, the guidelines were developed at different points in an evolving evidence base. The current RCOG PPH guideline predates the publication of the WOMAN Trial, while ACOG Practice Bulletin No. 183 was published in 2017 and SOGC Guideline No. 431 in 2022 [1,20,21]. The 2025 WHO consolidated guideline reflects evidence accumulated across successive updates to PPH guidance. WHO updated its recommendation for early IV TXA within three hours of birth in 2017 following publication of the WOMAN Trial [3,5]. More recent evidence, particularly the E-MOTIVE Trial, informed the development of a standardized first-response approach emphasizing objective blood-loss assessment and rapid, concurrent implementation of uterine massage, uterotonic therapy, TXA, IV fluids, genital tract examination, and escalation of care [3,13]. Thus, the evolution of WHO guidance reflects both the WOMAN Trial evidence supporting early TXA administration and subsequent evidence supporting its integration within coordinated first-response PPH management. Differences among these guidelines should therefore not necessarily be interpreted as disagreement regarding the efficacy of TXA. Rather, they partly reflect differences in the evidence available during guideline development and the subsequent incorporation of emerging evidence into clinical recommendations.

The timing of TXA remains particularly important. The WOMAN Trial demonstrated the greatest reduction in death due to bleeding when TXA was administered within three hours of delivery [5]. Given this time-dependent benefit, differences in how explicitly guidelines communicate treatment timing may represent an important implementation issue. However, no direct comparative evidence shows that implementing WHO, ACOG, SOGC, or RCOG guidance results in different maternal outcomes. Therefore, it would be inappropriate to conclude that one guideline framework is clinically superior based solely on differences in recommendation wording. Studies examining whether differences in treatment protocols influence time to TXA administration, transfusion requirements, invasive interventions, or maternal morbidity could help determine whether variation in guideline recommendations translates into clinically meaningful differences in practice.

The broader hemostatic context further supports distinguishing TXA from alternative therapies rather than considering these agents as competing interventions. Fibrinogen replacement, rFVIIa, and desmopressin have targeted roles determined by specific hemostatic abnormalities or clinical circumstances, while EACA lacks comparable randomized evidence in PPH [3,17-19]. Accordingly, the evidence supporting these therapies pertains to more specific clinical circumstances than the evidence supporting early TXA use in established PPH. Future research may therefore be more informative if it focuses on selecting targeted hemostatic therapies and combining them with early TXA based on hemorrhage severity, etiology, coagulation abnormalities, and response to treatment.

Current evidence also has implications for future guideline development. Greater consistency in recommendations regarding early TXA administration and the evidence-supported three-hour treatment window could reduce ambiguity across treatment pathways. Future guideline updates could also clarify TXA's role relative to other initial interventions, standardize dosing and repeat-dose instructions where evidence supports them, and clearly distinguish treatment of established PPH from prophylactic administration before hemorrhage develops. Uniformity should not require identical PPH algorithms across healthcare systems, as differences in resources, treatment availability, and clinical infrastructure remain important. Instead, more consistent evidence-based recommendations for TXA administration could provide clearer guidance while preserving flexibility for local implementation.

This review has several limitations. As a narrative review, it did not employ a systematic record-level screening process or formal risk-of-bias assessment. The guideline comparison was limited to selected major international and national organizations and therefore does not encompass all PPH guidance used worldwide. Furthermore, the guidelines evaluated were developed during different evidence eras, complicating direct comparisons because observed differences may reflect the evidence available at the time of guideline development rather than persistent differences in clinical approach. Evidence supporting alternative hemostatic therapies is also less extensive and more heterogeneous than that supporting TXA. Finally, the absence of direct comparative studies evaluating maternal outcomes under different guideline frameworks limits conclusions regarding the clinical consequences of the heterogeneity identified in this review.

## Conclusions

This review found broad agreement among major international guidelines on the role of TXA in PPH management, but differences in how they define its timing and position within treatment pathways. These differences should be considered in the context of evolving evidence and the different periods in which the evaluated guidelines were developed. Greater consistency across future guidelines may clarify TXA's role in PPH management while preserving flexibility for differences in healthcare settings, resources, and clinical practice. Important areas for further investigation include whether variation in guideline recommendations influences their implementation in clinical practice and whether these differences translate into meaningful differences in maternal outcomes.
